# Identification of the Optimal Cut-Off Value of PSA for Assessing Severity of Disease in [^68^Ga]Ga-PSMA-11 PET/CT Study in Prostate Cancer Patients after Radical Prostatectomy

**DOI:** 10.3390/diagnostics12020349

**Published:** 2022-01-29

**Authors:** Paulina Cegla, Marta Wojewódzka, Izabela Gorczewska, Wioletta Chalewska, Grażyna Łapińska, Paweł Ochman, Agata Sackiewicz, Marek Dedecjus

**Affiliations:** 1 Department of Endocrine Oncology and Nuclear Medicine, Maria Sklodowska-Curie National Research Institute of Oncology, 02-781 Warsaw, Poland; marta.wojewodzka@pib-nio.pl (M.W.); wioletta.chalewska@pib-nio.pl (W.C.); grazyna.lapinska@pib-nio.pl (G.Ł.); pawel.ochman@pib-nio.pl (P.O.); agata.sackiewicz@gmail.com (A.S.); marek.dedecjus@gmail.com (M.D.); 2 Department of Nuclear Medicine and Endocrine Oncology, Maria Sklodowska-Curie National Research Institute of Oncology, 44-102 Gliwice, Poland; izabela.gorczewska@io.gliwice.pl

**Keywords:** positron emission tomography/computed tomography, prostate-specific membrane antigen, prostate cancer

## Abstract

Objective: The objective of this study was to identify the optimal cut-off value of prostate specific antigen (PSA) to assess the extent of the disease in [^68^Ga]Ga-PSMA-11 PET/CT study in patients after radical prostatectomy. Materials and Methods: Retrospective analysis was performed on a group of 215 patients who underwent a [^68^Ga]Ga-PSMA-11 PET/CT examination because of suspected recurrence after radical prostatectomy. Patients were divided into four groups: 1, no active lesions suggesting recurrence (n = 92); 2, suspected isolated local recurrence (n = 19); 3, oligometastatic disease (n = 82); and 4, polymetastatic disease (n = 22). Results: In group 1, the mean PSA level was 0.962 ng/mL (median: 0.376; min: 0.004; max: 25 ng/mL); in group 2, it was 4.970 ng/mL (median 1.320; min: 0.003; max: 40.350 ng/mL); in group 3, it was 2.802 ng/mL (median: 1.270; min: 0.020; max: 59.670 ng/mL); and in group 4, it was 4.997 ng/mL (median: 3.795; min: 0.007; max 21.110 ng/mL). Statistically significant differences were shown in PSA levels when comparing groups 1 and 2 (*p* = 0.0025) and groups 3 and 4 (*p* = 0.0474). The PSA cut-off point for discriminating groups 1 and 2 was 0.831 (sensitivity: 0.684; specificity: 0.772; area under the curve (AUC): 0.775), and for groups 3 and 4, it was 2.51 (sensitivity: 0.682; specificity: 0.780; AUC: 0.720). Conclusions: Our preliminary data suggested that the PSA level has an essential influence on determining the extent of disease in a [^68^Ga]Ga-PSMA-11 PET/CT study in patients after radical prostatectomy. Identification of the optimal cut-off values for the oligo- and polymetastatic diseases might be helpful in stratifying these patients.

## 1. Introduction

Prostate cancer (PCa) ranks second in cancer diagnosis and fifth in cancer-related mortality in men worldwide [1]. The early stage of PCa is usually asymptomatic, and a biopsy, in combination with family history and prostate specific antigen level (PSA), is the only accurate diagnostic method to confirm or exclude PCa [2]. Patients with confirmed PCa generally have three treatment options: radical prostatectomy (RP), hormonotherapy (HT), or whole-gland radiation therapy (RT), which might be used alone or as part of a combined method [2]. Imaging in prostate cancer plays a pivotal role in staging and determining the extent of the disease. Among the available imaging modalities are magnetic resonance imaging (MRI), transrectal ultrasonography (TRUS), and positron emission tomography/computed tomography (PET/CT) with radiotracers showing avidity to the prostate gland, choline-based radiotracers ([^11^C]MET, [^18^F]FCH), [^18^F]fluciclovin) and radiolabeled prostate-specific membrane antigen (PSMA) ligands ([^68^Ga]Ga-PSMA, [^18^F]F-PSMA, [^99m^Tc]Tc-PSMA) [2,3,4].

[^68^Ga]Ga-PSMA has recently been the most commonly used radiotracer for staging and detecting the recurrence of prostate cancer. Sachpekidis et al. showed that a [^68^Ga]Ga-PSMA PET/CT study had a 96% and 71% detection rate in assessing the severity of the disease in staging and recurrent prostate cancer, respectively [5,6]. Determination of the extent of the disease based on non-invasive methods is of great interest in managing patients with prostate cancer.

We aimed to investigate the optimal cut-off value for PSA levels and the severity of the disease in [^68^Ga]Ga-PSMA PET/CT in patients after radical prostatectomy.

## 2. Materials and Methods

A retrospective analysis was performed on a group of 415 adenocarcinoma prostate cancer patients who were referred to the Department of Endocrine Oncology and Nuclear Medicine in Maria Sklodowska-Curie National Research Institute of Oncology in Warsaw between October 2017 and December 2019. Inclusion criteria were: (a) histopathologically proven prostate adenocarcinoma; (b) only radical prostatectomy treatment; (c) suspected biochemical progression before any other type of treatment; and (d) a PET/CT study within 1.5 weeks of an estimated PSA level. The exclusion criteria were: (a) radio- or chemotherapy before PET/CT study; (b) a PSA level estimated more than 1.5 weeks before or after a PET/CT study; and (c) histopathology different from adenocarcinoma. Finally, the analysis was performed on a group of 215 patients (mean age: 67 ± 8 years) with suspected recurrence, based on biochemical progression, and after radical prostatectomy.

Patients (after they gave their informed consent to the PET/CT study) underwent a [^68^Ga]Ga-PSMA-11 PET/CT study 60 min after intravenous injection of 172 ± 25 MBq according to the European Association of Nuclear Medicine (EANM) and Society of Nuclear Medicine and Molecular Imaging (SNMMI) procedure guidelines [7]. Images were acquired from the skull vertex to the mid-thigh on a Gemini TF TOF PET/CT (Philips Medical Systems, Cleveland, OH, USA) scanner with 2 min per bed position, following a low-dose CT scan (120 kV, 50 mAs) for attenuation correction. Because of the retrospective nature of the study, bioethical committee approval was not obtained.

The PSMA-11 kit (POLATOM, Otwock, Poland) was labeled with [^68^Ga]GaCl3 (^68^Ge/^68^Ga generator, GalliaPharm, Eckert–Ziegler, Berlin, Germany), and 600 MBq of eluate was added to the PSMA vial. After heating for 10 min at 95 °C, it was cooled for 10 min, and quality control was performed.

Radiochemical purity was assessed with the radioTLC (LabLogic, Scan-RAM, Broomhill, Sheffield, United Kingdom). The stationary phase used was ITLC-SG (Agilent Technologies, California, United States), and the mobile phase was a 10% solution of ammonium acetate in water/methanol 1:1 (*v*/*v*). The [^68^Ga]Ga-colloids retardation factor was 0.0–0.1, and the [^68^Ga]Ga-PSMA retardation factor was 0.9–1.0.

Patients were divided into 4 groups based only on [^68^Ga]Ga-PSMA-11 PET/CT results:

1. without any active lesions suggesting recurrence—42.8% (n = 92);
2. with suspected isolated local recurrence only in the gland—8.8% (n = 19);
3. with oligometastatic disease—38.1% (n = 82);
4. with polymetastatic disease—0.3% (n = 22).

Statistical analysis was performed on Statistica StatSoft Poland version 13.

The normality of data distribution was confirmed using the Shapiro–Wilk test, and, based on the result, a Mann–Whitney test was performed. Additionally, ROC analysis was performed to find the best cut-off point for the (mean) PSA level to determine the extent of the disease. A value of *p* < 0.05 was assumed to be significant.

In most of the patients, the TNM stage and the Gleason score were also assessed, and they were used for stratifying patients into low-, intermediate-, and high-risk. The TNM stage in 41 patients and Gleason score in 12 patients were not available for assessment.

## 3. Results

The [^68^Ga]Ga-PSMA-11 PET/CT study was performed after 34 ± 36 months after RP (range: 8–144 months). In the whole analyzed group, the mean PSA level was 2.43 ± 5.97 (ng/mL), while the mean values for all of the assessed groups are shown in Table 1.

The [^68^Ga]Ga-PSMA-11 study was positive in 57.2% of patients, whereas in 42.8% of patients, the study did not reveal any changes (Group 1).

The TNM stage and Gleason score for all patients divided into subgroups are shown in Table 2 and Table 3.

Even though it was assumed that a Gleason score from 6 indicated cancer, in the eight patients with a Gleason score lower than 6, adenocarcinoma prostate cancer was confirmed during histopathological examination from the tissue obtained from the radical prostatectomy. Among all groups, 12 patients did not have a Gleason score assessed: seven in group 1; two in groups 3 and 4; and one in group 1.

**Figure 1:**
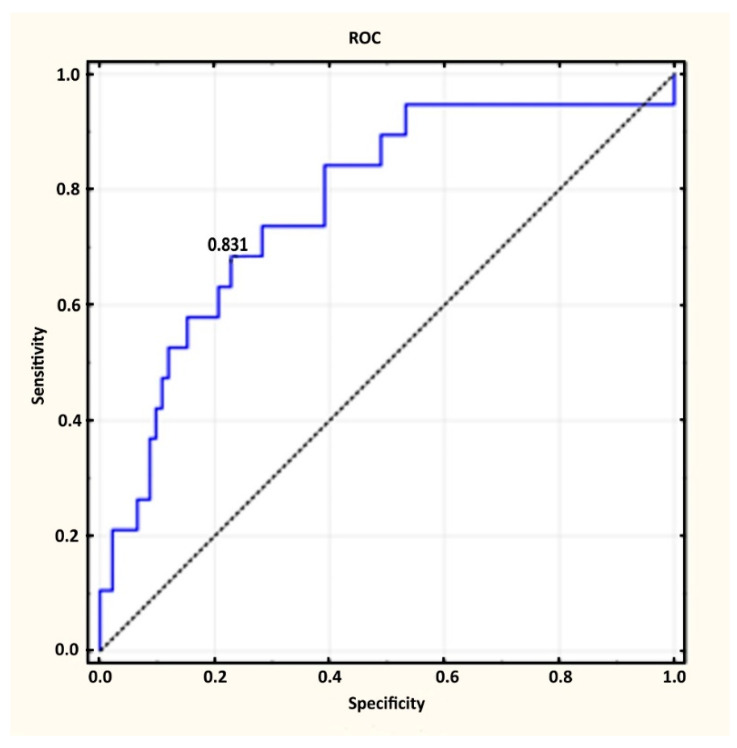
Suggested cut-off point to determine patients with or without isolated local recurrence.

To discern patients without any active lesions suggesting recurrence (group 1) from patients with isolated local recurrence (group 2) based on [^68^Ga]Ga-PSMA-11 results, the cut-off point was 0.831 ng/mL (specificity: 0.772; sensitivity 0.684; AUC: 0.775 (Figure 1)). In addition, based on the adopted level, α = 0.05 and *p* < 0.0001, it was found that the detection of recurrence using the PSA level was significantly more encouraging than the random division of patients into two groups: those without changes and those with suspected recurrence in the prostate fossa.

In group 1, 77.2% (n = 71) of patients had a PSA level below 0.831 and a negative [^68^Ga]Ga-PSMA-11 PET result, while in group 2, this accounted for only 36.8% (n = 7) of patients.

**Figure 2:**
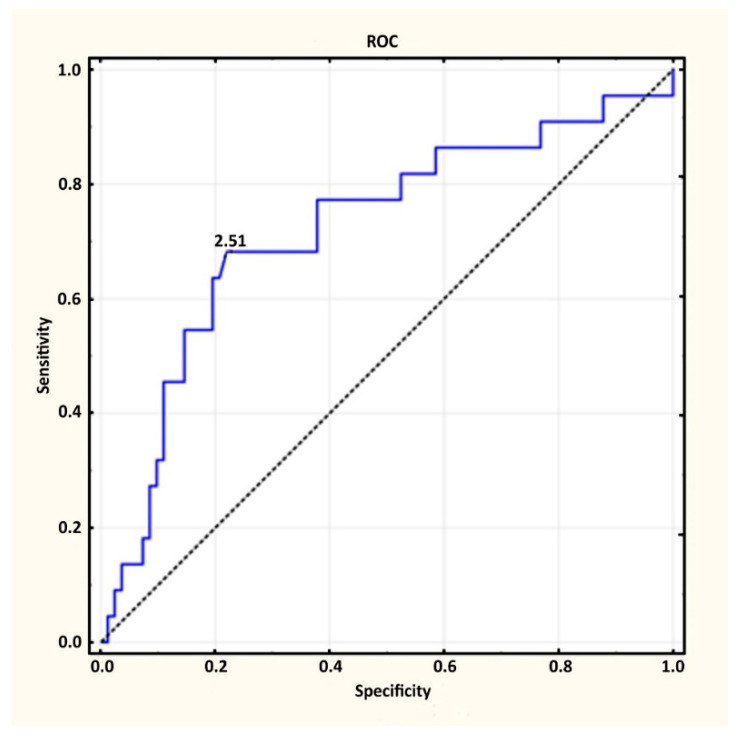
Suggested cut-off point to determine patients with oligometastatic and polymetastatic disease.

Performing a similar analysis to the discrepancy between groups 3 and 4, the best cut-off point based on [^68^Ga]Ga-PSMA-11 results was 2.51 ng/mL (specificity 0.780; sensitivity 0.682; AUC 0.72. (Figure 2)). Again, in the analysis performed using the ROC curve based on the adopted level, α = 0.05 and *p* = 0.0009, it was noted that detecting a local recurrence with PSA level was significantly better than dividing patients with oligometastatic and polymetastatic disease into two random groups.

In group 3, 70.7% (n = 65) of patients with oligometastatic disease had a PSA value below 2.51 ng/mL, while in group 4, there was only 31.8% (n = 7).

Similar to the ROC curve, a comparative analysis of group 1 with 2 and 3 with 4 was performed, looking for differences between the PSA level distributions. Statistically significant differences were shown in the PSA level between groups 1 and 2 (0.963 ± 2.779 vs. 4.970 ± 10.553; *p* = 0.0025) and between groups 3 and 4 (2.802 ± 6.965 vs. 4.997 ± 4.896; *p* = 0.0474).

## 4. Discussion

Nuclear medicine imaging is a safe and non-invasive technique used especially in patients with biochemical recurrence (BR), which is commonly assessed based on a rising PSA level [3]. Choline-based radiotracers have been used for years in PCa imaging; however, sometimes they have a limited value for identifying local recurrence, benign prostatic hyperplasia (BPH), and lymph nodes [8]. [^68^Ga]Ga-PSMA-11 has recently become the most commonly used radiotracer in PET/CT studies to evaluate prostate cancer patients [9], and, in comparison with conventional imaging techniques, it has the ability to detect small regions that might be helpful for assessing lymph node status when PSA levels are low [10].

Several authors compared the detection of BR based on a PSA level and different radiotracers in prostate cancer patients. In those with a PSA level lower than 1 ng/mL, the percentage of positive PET results was 7–44% for radiotracers labeled with choline, 21–41% with fluciclovine, and 29–67% with PSMA. In patients with a PSA level from 1 to 2 ng/mL, positive PET results were found in 25–81, 46–78, and 46–93% for choline-, fluciclovine- and PSMA-labeled radiotracers, respectively. When the PSA level was above 2 ng/mL, positive PET results were seen in 55–89% of patients with radiotracers labeled with choline, 55–86% with fluciclovine, and 71–97% with PSMA [11]. These data suggest that PSMA-labeled radiotracers show better diagnostic accuracy than other tracers, not only in patients with PSA below 1 ng/mL but also in patients with higher PSA values. Some authors confirmed this statement and even provided data to show that PSMA-based PET radiotracers were preferable to other radiotracers in a PSA level < 0.5 ng/mL [12].

Recently, Fendler et al. published an analysis of a group of 635 prostate cancer patients who underwent a [^68^Ga]Ga-PSMA-11 study in the case of biochemical recurrence. They concluded that a [^68^Ga]Ga-PSMA-11 examination showed a 84–92% positive predictive value (PPV) at a 75% overall detection rate (DR), with a median PSA level of 2.1 ng/mL. They also showed that when PSA was 0.5–1.0 ng/mL, the DR was 57% and improved significantly when PSA values increased [13]. However, they combined all of the patients after RP and after radiotherapy in one study, which might have had an influence on their results. In our study, in a comparison of a group of patients with no signs of metastasis in [^68^Ga]Ga-PSMA-11 and patients with isolated local recurrence, it was found that a cut-off point of 0.831 ng/mL with a specificity of 0.772 and moderate sensitivity of 0.684 made it possible to distinguish these patients. The suggested cut-off point in our study was higher than 0.2 ng/mL, which is the European Association of Urology (EAU) guideline for diagnosing biochemical recurrence in patients after a radical prostatectomy [14]; however, the [^68^Ga]Ga-PSMA-11 PET/CT study has recently become the most reliable imaging modality to allow confirmation or exclusion of PCa metastases and uses a cut-off point to distinguish patients, which might be helpful in qualifying them into appropriate treatment. Hoffmann et al., based on PSA values and ROC curves, estimated a cut-off point of 1.24 ng/mL (AUC: 0.784) for determining positive from negative [^68^Ga]Ga-PSMA-11 PET scans in patients after radical prostatectomy [15]. At this cut-off level, a [^68^Ga]Ga-PSMA-11 PET scan was positive in 52% of patients. Our study showed that using a level of 0.831 ng/mL and a similar AUC (0.775) allowed the prediction of a positive [^68^Ga]Ga-PSMA-11 result in 77.2% of patients. Nonetheless, differences in cut-off points and the number of positive PET scans in both studies (Hoffmann et al. and ours) showed significant differences between groups with and without metastases revealed during [^68^Ga]Ga-PSMA-11 study.

Some authors suggested that performing a [^68^Ga]Ga-PSMA-11 PET/CT study might have an impact on PCa patient management [16,17]. In their work on a group of 270 patients with a BR and PSA concentration less than 1.0 ng/mL, Calais et al. found that [^68^Ga]Ga-PSMA-11 had a major impact on salvage radiotherapy (SRT) planning in 19% of patients [16]. On the other hand, Bashir et al. in a smaller study (n = 28) found that [^68^Ga]Ga-PSMA-11 PET/CT had a high DR in assessing early BR, and only in one patient was polymetastatic disease found, whereas oligometastatic disease was diagnosed in seven patients. They assumed that [^68^Ga]Ga-PSMA-11 changed treatment management in 42.8% of patients [17].

Our analysis showed oligometastatic disease in 82 patients having a mean PSA value of 2.802 ng/mL (ranging from 0.020 to 59.670) and polymetastatic disease in 22 patients having a mean value of 4.997 ng/mL (ranging from 0.007 to 21.110), which was 48.4% of all patients included in the analysis. This was in concordance with abovementioned authors, who found that [^68^Ga]Ga-PSMA-11 had a large impact on patient management.

In their meta-analysis involving 16 papers with 1309 patients, Perera et al. showed that [^68^Ga]Ga-PSMA PET was positive in 76% of patients with BR and increased with pre-PET PSA [18].

Distinguishing PCa patients with local or locoregional recurrence from those with distant metastases plays a pivotal role in the proper choice of treatment. Increasing PSA levels are helpful for detecting PCa; however, they cannot determine whether it is a local, regional, or distant disease. Singh et al., in their study, noted that patients with fewer than five metastases had similar survival compared to those without and were significantly (*p* = 0.02) better compared to those with polymetastatic disease [19]. Even though we did not assess survival rate in our study, we found the cut-off point that might be helpful for distinguishing patients with oligo- and polymetastatic disease, and this may play an important role in the treatment options for these patients. The major limitation of [^68^Ga]Ga-PSMA is that it is eliminated in the urine, which causes high bladder activity and problems with detecting local recurrence among PCa patients [20,21]. Another PSMA tracer ([^18^F]PSMA-1007) has been shown to overcome this limitation, because it is mainly excreted through the liver, with only 1–2% of the injected activity seen in the urine [22]. This gives an opportunity to detect PSMA-positive lesions in local recurrence near the urinary tract [22].

Several authors indicated that dynamic imaging in [^68^Ga]Ga-PSMA might also be helpful to differentiate between pathologic uptake and physiological bladder accumulation [23]. Recently, Strauss et al. performed a retrospective analysis on a group of 142 patients with BR [24] and found that, even in patients with a low PSA level (between 0.55 and 0.99 ng/mL), the detection rate was 57%. Moreover, they also suggested that when dynamic acquisition was performed within the first 10 min post-injection, the tumor showed high activity compared to the non-measured activity in the bladder [24]. This solution provides a better diagnosis of detection of local recurrence near the bladder [6].

Among all PCa, around 5–10% are PSMA-negative on immunohistochemical PSMA expression, and, of those, about 10% give a negative PSMA-PET result, in spite of a rising PSA level [25]. Some authors suggest that, even though PSMA-negative tumors have a better prognosis, the detection of recurrence in this type of cancer patient is challenging, because it gives a false negative PSMA-PET result [26]. This might also explain why, for some patients from our group 1, the [^68^Ga]Ga-PSMA PET scan was negative despite a high PSA level, the maximum of which was 25 ng/mL.

Recently, Evangelista et al. investigated the value of several imaging modalities (including CT, MRI, PET, and PET/MRI) in patients with oligometastatic recurrence of prostate cancer [27]. They indicated that standard CT imaging as well as whole-body scintigraphy (WBS) were not sensitive enough compared to a multiparametric MRI (mpMRI) [28]. It was shown that the overall DR for MRI was 65.7% and 37.1% for PET, while for PET/MRI, it was 74.3% [28]. Freitag et al. explored the additional value of mpMRI compared to the [^68^Ga]Ga-PSMA-11 PET on a group of 119 patients with biochemical recurrence [29]. Both studies indicated that PET/MRI with mpMRI provided additional value and diagnostic information compared to other imaging modalities in the detection of local recurrence.

Our study had several limitations. Firstly, it was a single-center retrospective analysis, and the PSA level was not assessed at the time of [^68^Ga]Ga-PSMA PET examination but within 1.5 weeks. Secondly, histopathological confirmation of the recurrence in lymph nodes and prostate fossa was not assessed. Thirdly, the initial PSA level was not assessed, and there was a limitation due to the method used, because micrometastases (<5 mm) might have been missed. Moreover, a high percentage (about 20%) of patients had no pTNM determination. Lastly, no follow-up data or overall survival were assessed. Nevertheless, despite these limitations, this study confirmed that the PSA level had an influence on [^68^Ga]Ga-PSMA results and that a further, prospective, multi-center study with histopathological confirmation of findings is needed.

## 5. Conclusions

Our preliminary data suggested that PSA level has an essential influence on determining the extent of disease in a [^68^Ga]Ga-PSMA-11 PET/CT study in patients after radical prostatectomy. Identification of the optimal PSA cut-off level based on the [^68^Ga]Ga-PSMA-11 study might be helpful for stratifying these patients. Further larger studies on a more homogeneous group are warranted.

## Figures and Tables

**Table 1:** Mean values for PSA level in the analyzed groups.

Group	Mean (ng/mL)	Median (ng/mL)	Min (ng/mL)	Max (ng/mL)	SD
1	0.963	0.376	0.004	25.000	2.779
2	4.970	1.320	0.003	40.350	10.553
3	2.802	1.270	0.020	59.670	6.965
4	4.997	3.795	0.007	21.110	4.896

**Table 2:** Characteristics of TNM stage.

TNM Stage	All Groups	Group 1	Group 2	Group 3	Group 4
T1cN0	3	2	1		
T2	7		3	2	2
T2aN0	13	8	1	3	1
T2bN0	15	5		10	
T2bN1	3		1	2	
T2cN0	44	23	4	14	3
T3aN0	32	13	2	14	3
T3aN1	3	2		1	
T3aN0M1b	3	1		2	
T3bN0	35	14	2	18	1
T3bN1	13	6		3	4
T4N0	1	1			
T4N1	2		1	1	

**Table 3:** Characteristics of Gleason score.

Gleason Score	All Groups	Group 1	Group 2	Group 3	Group 4
2	1		1		
4	1		1		
5	6	2	1	2	1
6	31	17	5	7	2
7	96	41	7	39	9
8	33	14	1	14	4
9	32	15	2	11	4
10	3	1		2	

## Data Availability

The data presented in this study are available on request from the corresponding author. The data are not publicly available due to legal requirements of data protection.
